# The Gut Microbiome in Sleep Disorders: A Review of Recent Evidence

**DOI:** 10.62641/aep.v54i2.2123

**Published:** 2026-04-15

**Authors:** Alejandro Borrego-Ruiz, Juan J. Borrego

**Affiliations:** ^1^Departamento de Psicología Social y de las Organizaciones, Universidad Nacional de Educación a Distancia (UNED), 28040 Madrid, Spain; ^2^Departamento de Microbiología, Universidad de Málaga, 29071 Málaga, Spain

**Keywords:** sleep disorders, sleep regulation, gastrointestinal microbiome, synbiotics, fecal microbiota transplantation

## Abstract

Alterations in the gut microbiome have been shown to influence sleep through gut-brain interactions. However, the interplay between the gut microbiome and sleep disorders remains insufficiently understood. This narrative review provides an overview of recent evidence on the role of the gut microbiome in sleep disorders, examining host-microbial regulation of the sleep cycle, the relationship between gut microbiome dysbiosis and sleep disorders, the influence of the gut microbiome on sleep-related breathing disorders, sleep deprivation, and sleep fragmentation, as well as microbial therapeutic approaches to sleep disorders. Through its effects on bacterial metabolites, immune responses, and neuronal signaling, the gut microbiome might be potentially involved in the regulation of sleep-wake cycles. Disturbances in sleep have been associated with shifts in gut microbiome composition, but this relationship remains incompletely understood and it suggests a bidirectional nature. Evidence indicates that interventions targeting the gut microbiome, such as the use of psychobiotics and fecal microbiota transplantation, may have potential for improving sleep outcomes, but further research is needed to determine their actual effectiveness. Understanding the full range of factors influencing the gut microbiome and their interactions with other variables will be essential for elucidating the mechanisms behind gut-sleep interactions. Thus, future studies should focus on clarifying causality, identifying key biomarkers, and developing microbial-based interventions to establish effective therapeutic strategies.

## Introduction

Sleep constitutes a reversible physiological process that involves specific patterns of cerebral electric activity and is maintained through a complex interplay of neurotransmitters and neuromodulators within the central nervous system [1]. Living organisms possess an intrinsic sleep-wake cycle synchronized with the 24-hour alternation of light and darkness. This process is governed by the circadian rhythm, which plays a pivotal role in metabolic regulation, hormonal balance, and the maintenance of overall physiological homeostasis [2]. Sleep is categorized into two distinct states: non-rapid eye movement (NREM) sleep and rapid eye movement (REM) sleep, each contributing differently to health and neural processing. NREM sleep is primarily associated with somatic recovery, including tissue regeneration and immune enhancement, whereas REM sleep is closely linked to cognitive processes such as dreaming and memory consolidation. NREM sleep itself progresses through three sequential stages: NREM1 represents the transition from wakefulness to sleep; NREM2 constitutes a phase of light sleep; and NREM3 corresponds to deep or slow-wave sleep, which is subsequently followed by entry into REM sleep [1, 2].

Sleep disorders can be understood as networks of interconnected symptoms in which certain central symptoms, such as insomnia or daytime sleepiness, can trigger or maintain other symptoms within the symptom network, with objective markers, patient-reported experiences, and symptom interactions defining the pathological profile [3]. Sleep disorders are heterogeneous in pathogenesis and manifestation. According to the last version of the International Classification of Sleep Disorders (ICSD-3-TR), main conditions include insomnia disorder, central disorders of hypersomnolence, sleep-related breathing disorders, circadian rhythm sleep-wake disorders, parasomnia disorders, and sleep-related movement disorders [3]. Thus, sleep is closely linked to mental health, with factors such as altered daily routines, anxiety, reduced daylight exposure, social isolation, and stress contributing to sleep disturbances [4]. In turn, sleep disturbances negatively affect cognitive functioning, which can result in disrupted daily life and in mental health conditions, such as insomnia and emotional disorders [4]. Moreover, inconsistent sleep patterns have become increasingly prevalent and these sleep irregularities have been associated with a range of health consequences, such as cardiovascular disease, diabetes, metabolic syndrome, obesity, and mortality [5]. Neurobiological mechanisms and psychosocial variables have been suggested as central mediators of the relationship between sleep disturbances and mental health [4]. Specifically, disrupted prefrontal cortex activity, altered hypothalamic-pituitary-adrenal (HPA) axis function, immune-inflammatory system impairments, personality traits, and both demographic and environmental factors have been proposed as potential modulators of this relationship [4, 6].

Over the past few years, the gut-brain axis (GBA) has gained attention as a promising framework for explaining how sleep disturbances may interact with psychiatric pathology [7]. Disruptions in the gut-brain barrier, particularly alterations in the gut microbiome (GM), have been shown to influence sleep and mental states through microbiota-gut-brain interactions [7]. Indeed, evidence implicates the GM as a potential mediator of both acute and chronic health consequences arising from insufficient or fragmented sleep [8]. While the GM impacts sleep physiology, disruption in sleep patterns can also alter the composition and function of the GM [8]. In this context, interventions targeting the GM have demonstrated promise in improving sleep quality, suggesting potential therapeutic tools for sleep disorders [8]. Despite growing interest, the interplay between the GM and sleep disorders remains insufficiently understood. In order to address this gap, the present narrative review provides an overview of recent evidence on the role of the GM in sleep disorders by examining: (i) host-microbial regulation of the sleep cycle; (ii) the relationship between GM dysbiosis and sleep disorders; (iii) the influence of the GM on sleep-related breathing disorders, as well as on sleep deprivation and sleep fragmentation; and (iv) microbial therapeutic approaches to sleep disorders.

## Methods

A non-systematic, narrative approach was employed to conduct the review, with the primary aim of providing an overview of recent evidence regarding the role of the GM in sleep disorders. Literature searches were conducted between August and September 2025, using the PubMed, Scopus, and Web of Science databases. In addition, reference lists from relevant articles were reviewed to identify potentially significant studies. No restrictions were placed on language, but only articles published between 2020 and 2025 were considered. The search strategy followed an iterative process, incorporating a variety of keywords related to the topic under examination, such as “sleep”, “sleep disorders”, “sleep disturbances”, “sleep regulation”, “sleep cycle”, “sleep-related breathing disorders”, “insomnia”, “sleep deprivation”, “sleep fragmentation”, “circadian rhythms”, “gut microbiome”, “microorganisms”, “dysbiosis”, “psychobiotics”, “probiotics”, “prebiotics”, “synbiotics”, and “fecal microbiota transplantation”. The selection process consisted of two main phases. First, articles were screened based on their titles and abstracts, with studies deemed irrelevant being excluded. Second, the full texts of articles that passed the initial screening were fully reviewed, and those failing to meet the inclusion criteria were discarded. Inclusion criteria encompassed studies that examined the influence of the GM in sleep and related conditions, the relationship between GM dysbiosis and specific sleep disturbances, and microbial therapeutic approaches to sleep disorders. Exclusion criteria encompassed studies that did not provide pertinent information related to the aim of the review, as well as theses, letters, and conference abstracts. Relevant data from each article were extracted, synthesized, and organized into thematic sections for the review. Given its narrative and descriptive nature, no formal quality assessment of the selected studies was performed.

## The Gut Microbiome in Sleep Disorders

### Host-Microbial Regulation of the Sleep Cycle

Waking and sleep states alternate naturally between periods of wakefulness and the stages of REM and non-REM sleep [9]. These processes are governed by neurochemical changes involving neurotransmitters and neuromodulators, such as glutamate, acetylcholine (ACh), γ-aminobutyric acid (GABA), norepinephrine (NE), dopamine (DA), serotonin (5-HT), histamine, hypocretin, adenosine, and melatonin, which act through complex interactions within neural networks to regulate sleep-wake cycles [9]. Several of these signaling substances, including 5-HT, ACh, DA, GABA, melatonin, and NE, are also synthesized or metabolized by the GM, thereby playing a role in modulating sleep processes [10]. In turn, gut microorganisms exhibit rhythmic activities influenced by factors such as diet, and disruption in sleep can change the composition of the GM, indicating its contribution to microbial balance [10]. Thus, the GM regulates sleep-wake behavior via modulating bacterial metabolites, endocrine signaling, neuronal signaling, and immune responses [10]. For instance, *Bifidobacterium* and *Lactobacillus* species have the capacity to convert glutamate into GABA, thereby engaging GABAergic signaling mechanisms implicated in sleep regulation [11]. Similarly, *Clostridium sporogenes* facilitates the conversion of tryptophan (Trp) into 5-hydroxytryptophan via a Trp decarboxylase gene, subsequently promoting 5-HT synthesis [12]. In addition, recent findings indicate that microbial metabolic activity is essential for the neuroprotective effects of melatonin in models of cognitive impairment induced by sleep deprivation in mice [13]. The proposed mechanism suggests that sleep deprivation leads to reduced levels of *Lachnospiraceae* NK4A136 and butyrate in the colon. On the other hand, increases in *Aeromonas* abundance and lipopolysaccharide (LPS) accumulation contribute to inflammatory responses [13]. These changes, however, were reversed by melatonin supplementation, which also decreased *Aeromonas* and LPS levels [13]. High levels of pro-inflammatory molecules in the systemic circulation can also threaten the permeability of the blood-brain barrier, diminishing its ability to block LPS and inflammatory cytokines. LPS that enters the brain binds to Toll-like receptor 4 on microglia, triggering the synthesis and release of pro-inflammatory cytokines. These cytokines, in turn, activate specific neurons that project to key sleep-regulating areas such as the hypothalamus and brainstem, modulating the balance between wake-promoting and sleep-promoting neuron populations [13].

Microbial-derived compounds present in the gastrointestinal tract are capable of eliciting a sustained immune response, which can amplify local inflammatory signaling. Such inflammation engages microglial cells within the enteric nervous system (ENS), which simultaneously interact with vagal neural pathways [10]. Microorganisms exert continuous influence on immune cells through both direct and indirect mechanisms. Direct activation occurs via recognition of microbial-associated molecular patterns by pattern-recognition receptors, including Toll-like receptors and NOD-like receptors. Indirect modulation stems from microbial-derived metabolites that influence immune signaling pathways. The most common microbial metabolites are short-chain fatty acids (SCFAs), Trp derivatives, secondary bile acids and derivatives, histamine, sphingolipids, polyamines, p-cresol, and co-metabolites [14]. Consequently, immune cells serve a dual function, regulating both host-microbiota interactions and circadian rhythm mechanisms. The GM can directly influence the transcription of key circadian genes, such as Rev-ERBA (encoded by the *NR1D1 *gene) and Nfil3 (encoded by the *Nfil3 *gene), via the DC-ILC3-STAT3 immune pathway [15]. At the same time, microbial metabolites and structural components of bacterial cell walls, including LPS, interact with microglial cells within the innate immune system of the ENS, triggering an inflammatory cascade in the gut [14]. Notably, LPS derived from Gram-negative bacteria can markedly reduce electroencephalogram theta power, prolong NREM sleep, and shorten REM sleep, collectively contributing to increased host fatigue [16].

In summary, various factors have been identified as potential contributors to the regulation of sleep cycles: (i) hormonal signals such as melatonin, cortisol, leptin, and ghrelin; (ii) pro-inflammatory cytokines, which enter the brain via circumventricular organs or signal through the vagus nerve to activate central sleep circuits; (iii) metabolic signals such as adenosine; (iv) sensory inputs, including light, temperature, and sound; and (v) vagal nerve signaling. These peripheral inputs converge on and modulate the activity of core sleep-regulating circuits, particularly those involving the ventrolateral preoptic area, which is known to promote sleep, and wake-promoting systems such as the tuberomammillary nucleus, locus coeruleus, and dorsal raphe nucleus. The interaction of these signals ultimately determines whether the brain enters a state of sleep or wakefulness.

### The Relationship Between Gut Microbiome Dysbiosis and Sleep Disorders

Research has highlighted a bidirectional relationship between the GM and sleep disorders [10]. Disruption of circadian rhythms can lead to GM dysbiosis, affecting GM composition and metabolism [17]. Holzhausen *et al*. [18] provided evidence linking sleep parameters to GM composition. The study revealed notable within-person variations in the GM using three widely employed measures of microbial α-diversity (i.e., the diversity of GM composition within an individual). Moreover, the study reported that greater night-to-night variability in sleep duration and increased wake-after-sleep-onset (WASO) were significantly associated with reduced GM richness and diversity. Regarding β-diversity (i.e., the differences in GM composition between individuals), significant associations were found regarding habitual napping (at least once per week), self-reported sleep quality, sleep efficiency, and sleep latency, with night-to-night sleep duration variability showing the strongest correlation. Specific bacterial taxa, including members of the *Christensenellaceae* and *Mogibacteriaceae* families, were linked to higher sleep efficiency, improved self-reported sleep quality, longer average sleep latency, and greater night-to-night sleep duration variability. In a separate study focusing on older men, certain butyrate-producing bacteria, such as *Coprococcus*, were associated with increased sleep latency, a parameter often indicative of poorer sleep quality [19].

Accumulating evidence points to a potential role of GM alterations in shaping sleep architecture and contributing to sleep disorders [20]. A proposed view is that a highly diverse microbial profile is essential for maintaining physiological homeostasis, while reduced microbial diversity is associated with GM dysbiosis and various metabolic disturbances, including impaired sleep [21]. In this context, both sleep disorders and GM dysbiosis have been shown to increase the risk of metabolic conditions such as cardiovascular diseases (e.g., myocardial infarction, stroke), diabetes, and obesity [21]. Furthermore, sleep disruptions in older adults have been shown to drive changes in GM composition, with shorter sleep duration linked to an increase in pro-inflammatory bacteria, while improved sleep quality linked to higher levels of beneficial Verrucomicrobiota and Lentisphaerae phyla [22]. Building on these insights, it is conceivable that gut microorganisms and their metabolites may influence human behavior, the regulation of sleep, and the onset and progression of mental health conditions [21, 23].

**Fig. 1.**
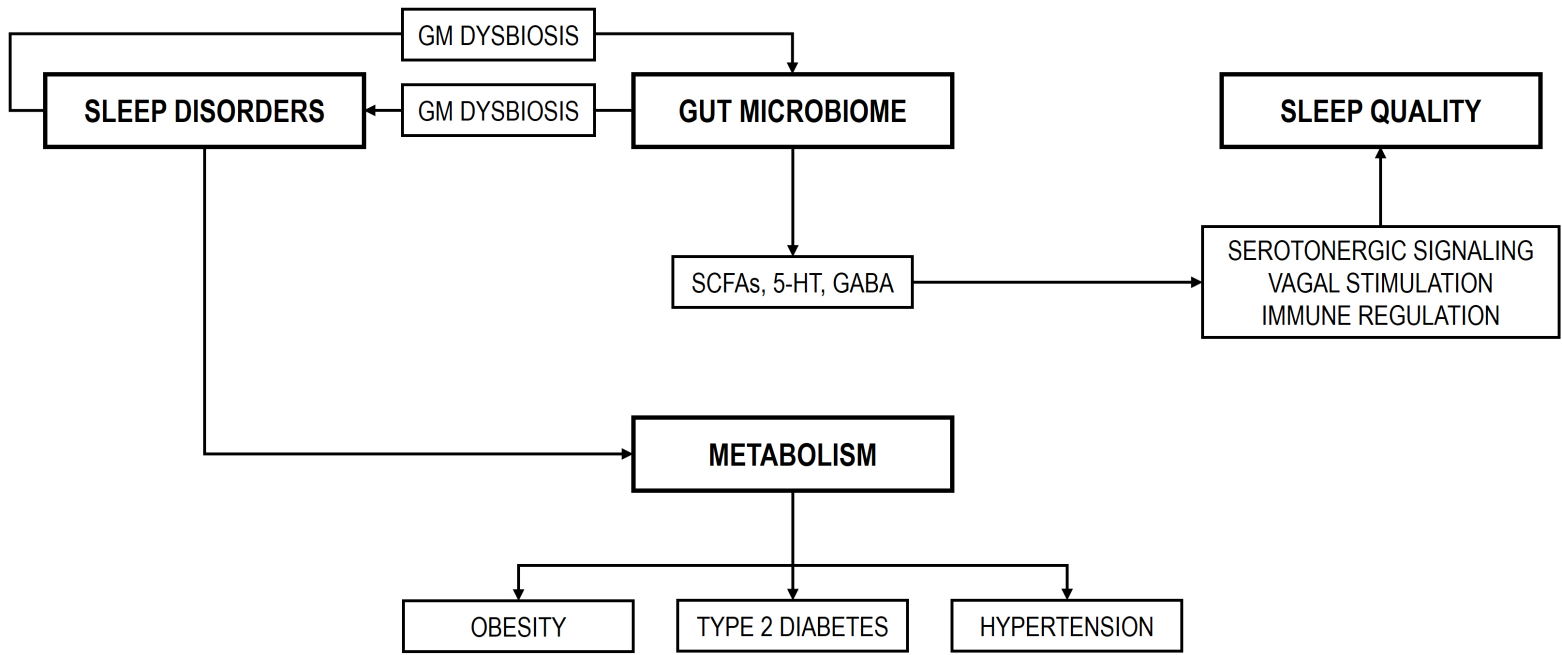
**Hypothetical representation between sleep disorders, the GM, and sleep quality**. SCFAs, short-chain fatty acids; 5-HT, serotonin; GABA, γ-aminobutyric acid; GM, gut microbiome.

A recent bidirectional Mendelian randomization (MR) study examined causal relationships between 119 bacterial genera and seven sleep-related traits [24]. In the forward MR analysis, inverse-variance weighted estimates indicated that the genetically predicted relative abundances of 42 bacterial genera exerted causal effects on sleep traits. Conversely, in the reverse MR analysis, sleep traits were found to causally influence 39 bacterial genera, 13 of which overlapped with those identified in the forward analysis. Specifically, genetically predicted abundances of *Holdemanella* and *Ruminococcaceae UCG-002* were negatively associated with daytime napping, while seven other genera, including* Butyricimonas*,* Defluviitaleaceae UCG-011*,* Eisenbergiella*,* Lachnospiraceae UCG-010*,* Oxalobacter*,* Ruminococcaceae UCG-013*, and *Ruminococcus gnavus* group, were positively correlated with this trait. Daytime sleepiness was positively linked to 11 genera, including *Alloprevotella*,* Butyricimonas*,* Clostridium sensu stricto 1*,* Collinsella*,* Coprococcus 2*, *Coprococcus 3*, *Eubacterium eligens* group,* Oxalobacter*, *Peptococcus*, *Ruminococcus gnavus* group, and *Slackia*. Regarding insomnia, positive associations were observed with *Clostridium innocuum* group,* Lachnoclostridium*,* Marvinbryantia*, *prevotella 7*, and *Rikenellaceae RC9* group, whereas *Odoribacter* and *Oscillibacter* showed negative correlations. In turn, *Alistipes* and *Eubacterium hallii* group were negatively associated with total sleep duration, whereas *Anaerofilum*,* Lachnospiraceae UCG-004*,* Odoribacter*,* Oscillibacter*, and *Victivallis* showed positive correlations. In addition, *Alistipes*,* Butyricimonas*, and *Ruminococcaceae NK4A214* group were inversely associated with long sleep duration, whereas* Ruminiclostridium 6* and *Slackia* displayed positive associations. For short sleep duration,* Anaerofilum*, *Coprococcus 1*,* Eubacterium fissicatena* group, *Lachnospiraceae UCG-004*, and *Oscillibacter* were negatively correlated, whereas* Barnesiella*, *Collinsella*, and *Eubacterium hallii* group exhibited positive correlations. Fig. 1 presents a hypothetical representation of the relationship between sleep disorders, the GM, and sleep quality.

### The Influence of the Gut Microbiome on Sleep-Related Breathing Disorders

Research has explored the relationship between GM dysbiosis and specific sleep disturbances in human populations. In a pilot study of pediatric obstructive sleep apnea (OSA) syndrome, Valentini *et al*. [25] reported increased Bacillota/Bacteroidota ratios, higher abundance of inflammation-associated bacterial strains, and reduced microbial diversity compared to controls. Notably, these microbial changes correlated with sleep parameters. Specifically, IL-6 concentrations were positively associated with both microbial diversity and specific members of Pseudomonadota, as well as with measures such as total sleep time and time spent in bed. More recently, Li *et al*. [26] found that there was no significant difference in Bacillota, Bacteroidota, Actinomycetota, and Pseudomonadota between patients with OSA and the controls after comparing the gut samples. After comparing the salivary samples of OSA patients with healthy controls, other study found that *Prevotella*, *Actinomyces*, *Bifidobacterium*, *Escherichia*, and *Lactobacillus* were enriched in the OSA group [27]. However, another report revealed that the relative abundances of *Prevotella*, *Veillonella*, *Bacteroides*, *Alloprevotella*, and *Leptotrichia* in the oral microbiota of patients with severe OSA were significantly lower than those in the healthy controls [28]. The contradictory results regarding the abundance of the genus *Prevotella* may be explained by differences between the two studies [27, 28] in sample types (i.e., salivary vs. global oral cavity), populations (i.e., children vs. adults), and methodologies (i.e., 16S rRNA gene sequencing vs. whole-genome metagenomics analysis). Furthermore, the genus *Prevotella* consists of multiple species, but the studies did not report on the presence or absence of the different species in the samples. Overall, all these findings suggest a potential association between the GM and OSA. However, they do not provide definitive evidence of causality. Conventional observational studies are limited in their ability to establish causal relationships due to the inherent risks of bias, reverse causality, and confounding factors. To better understand the connection between OSA and GM dysbiosis, large-scale cohort studies are necessary. Moreover, it remains unclear whether treating OSA can restore GM balance, or if GM-targeted interventions can effectively address OSA symptoms.

### The Influence of the Gut Microbiome on Sleep Deprivation and Sleep Fragmentation 

**Fig. 2.**
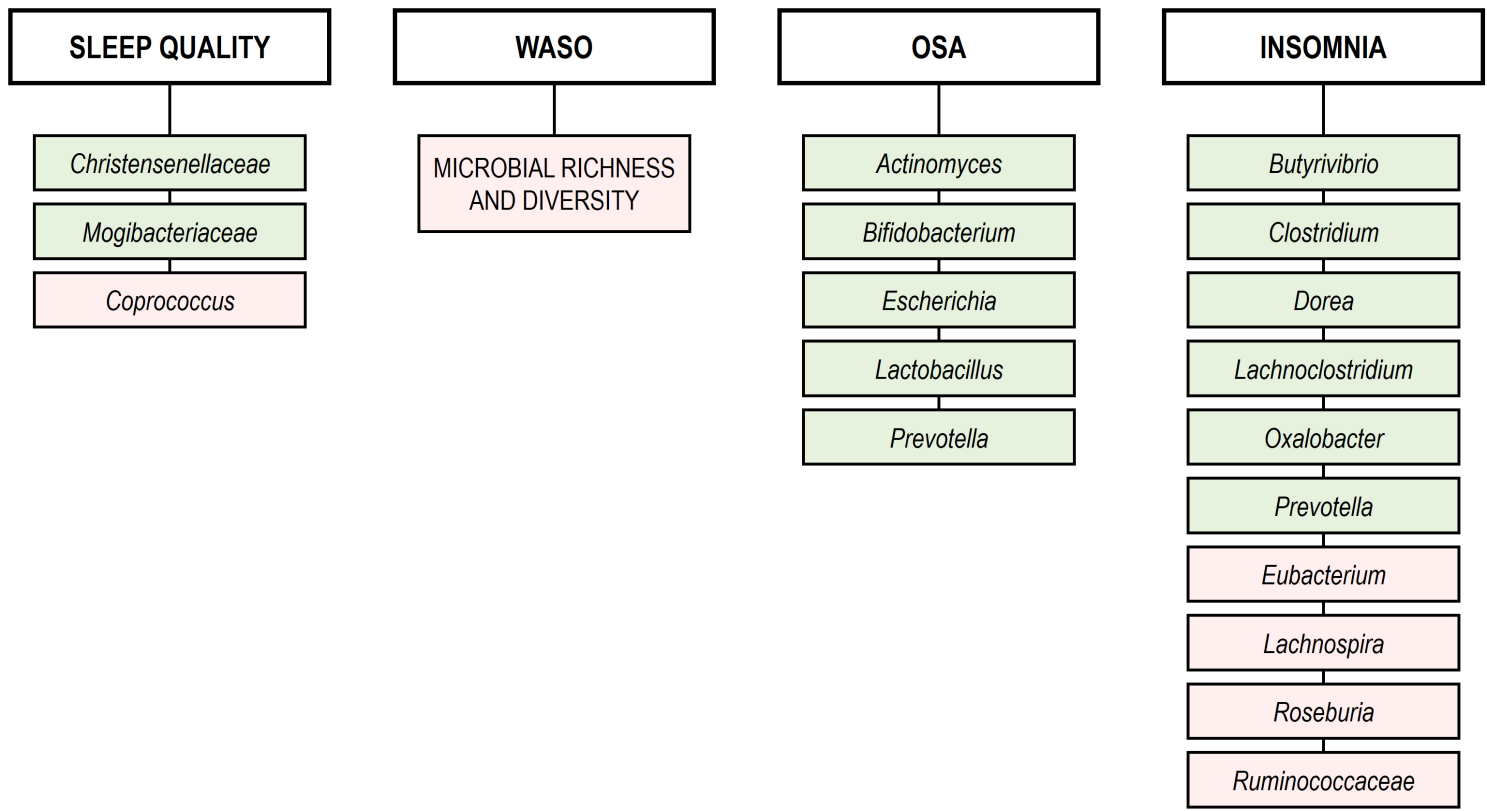
**GM dysbiosis linked to various sleep disorders**. WASO, wake after sleep onset; OSA, obstructive sleep apnea. Green rectangles: increase. Red rectangles: decrease. Microbial richness and diversity were assessed by determination of α-diversity (Chao1, Shannon, and Simpson indices) and β-diversity using the Bray-Curtis dissimilarity test (confidence level *p* < 0.05). Changes in microbial abundance (increase/decrease) were analyzed using 16S rRNA gene sequencing, LEfSe, and MR-Egger methods at *p* < 0.05.

Sleep deprivation refers to the condition of insufficient sleep, whether induced experimentally, caused by life events, or resulting from various pathophysiological factors, including medication effects, chronic illness, and psychiatric disorders. Research conducted in humans suggests that partial or prolonged sleep deprivation can alter GM composition [29, 30, 31]. Li *et al*. [29] identified ten GM taxa with causal associations to insomnia. Among these, *Clostridium innocuum*, *Dorea* spp., *Lachnoclostridium* spp., *Prevotella 7*, and the order Selenomonadales were linked to an increased risk of insomnia. In reverse MR analyses, insomnia was found to causally influence six additional GM taxa, increasing the abundance of* Butyrivibrio*,* Clostridium sensu stricto 1*,* Oxalobacter*, and *Oxalobacteraceae*, while decreasing the abundance of *Eubacterium nodatum* group and *Ruminococcaceae UCG-013* [29]. Consistent with these findings, patients with acute or chronic insomnia exhibit reduced levels of several anaerobic gut microorganisms, including *Faecalibacterium*, *prevotella 9*, and *Roseburia* [30]. Interestingly, Karl *et al*. [31] reported that severe, short-term sleep restriction reduced GM richness in healthy young men without affecting intestinal permeability. In contrast, Wang *et al*. [32] observed that 40 hours of total sleep deprivation in healthy adults led to alterations in GM composition and also to increased circulating markers of HPA-axis activation, inflammation, and intestinal permeability. These discrepancies may reflect differences in study populations, experimental designs, or suggest that the effects of sleep restriction on the human GM depend on the duration and severity of sleep loss. In a study examining patients with psychiatric disorders, Mairinger *et al*. [33] analyzed GM composition in relation to sleep. Although no significant correlations were observed between microbial diversity and Pittsburgh Sleep Quality Index (PSQI) scores in either patients or controls, certain taxa were differentially abundant among psychiatric patients with good sleep quality (PSQI >8) compared to those with poor sleep quality (PSQI ≤8). These included three species (i.e., *Ellagibacter isourolithinifaciens*, *Senegalimassilia faecalis*, and uncultured *Blautia* spp.) and two genera (i.e., *Senegalimassilia* and uncultured *Muribaculaceae*). Furthermore, research in mice has reported comparable findings. In this respect, a recent study demonstrated that five days of sleep interruption significantly affected GM composition and metabolomic profiles, reducing levels of beneficial bacteria, altering microbial metabolic functions, and modifying fecal concentrations of bacterial metabolites [34]. Fig. 2 presents the main features of GM dysbiosis linked to various sleep disorders.

## Microbial Therapeutic Approaches to Sleep Disorders

Interventions targeting the GM have demonstrated therapeutic potential across multiple psychiatric and brain-related disorders [35]. Given the side effects of conventional sleep medications, alternative or complementary treatment strategies for sleep disorders may be valuable in clinical practice. In this context, GM-targeted approaches such as psychobiotics or fecal microbiota transplantation (FMT) could constitute promising candidates for the management of sleep-related conditions as adjunctive therapies.

Psychobiotics represent an emerging class of psychotropic agents, encompassing both live microorganisms and bioactive compounds that confer beneficial effects in individuals with mental health conditions [35]. Specifically, psychobiotics encompass probiotics, prebiotics, and synbiotics that are employed to alleviate neuropsychiatric symptoms [35]. According to Mörkl *et al*. [36], the primary mechanisms through which psychobiotics exert their effects include: (i) modulation of the HPA axis; (ii) synthesis of key neurotransmitters; (iii) regulation of brain-derived neurotrophic factor; (iv) influence on oxytocin signaling; (v) interaction with the vagus nerve; (vi) production of postbiotics; (vii) maintenance and enhancement of intestinal barrier integrity; (viii) immunomodulatory effects; (ix) suppression of pathogenic microorganisms; and (x) shaping and refinement of neural networks.

Probiotics are live microorganisms that, when administered in adequate amounts on a regular basis, confer measurable health benefits to the host. They contribute to the maintenance of a balanced GM, support the regeneration of intestinal mucosal cells, activate the vagus nerve, and promote proper immune function [35]. Many of these effects are mediated, at least in part, by the production of SCFAs such as acetate, butyrate, and propionate, which play a pivotal role in regulating intestinal homeostasis, energy metabolism, colonocyte function, and immune responses [35]. For instance, the intervention with the probiotic *Lactiplantibacillus *(formerly *Lactobacillus*)* plantarum *JYLP-326 appears to alleviate symptoms of anxiety, depression, and insomnia, potentially by modulating GM composition and by altering fecal metabolite profiles, which are significantly associated with anxiety-related symptoms [37]. Moreover, probiotics such as *L. plantarum *PS128, *Lactobacillus gasseri *CP2305, *Lacticaseibacillus acidophilus *Rosell-53, *Bifidobacterium longum *Rosell-175, *Limosilactobacillus reuteri *NK33, *Bifidobacterium adolescentis *NK98, *L. reuteri *PBS072, and *Bifidobacterium breve *BB077 have been shown to improve sleep quality and alleviate sleep disturbances [35].

Prebiotics are non-digestible dietary components that selectively stimulate the growth and activity of beneficial gut microorganisms. They include: (i) carbohydrates such as fructooligosaccharides (FOS), galactooligosaccharides (GOS), inulin, oligosaccharides, and resistant starch; (ii) phytochemicals such as chlorogenic acids, epigallocatechin gallate, quercetin and resveratrol; and (iii) polyunsaturated fatty acids such as docosahexaenoic acid, eicosapentaenoic acid, and omega-3 [38]. Same as with probiotics, these compounds promote host health partly by enhancing the release of SCFAs, thereby contributing to gut homeostasis and systemic physiological benefits [38, 39]. Regarding mental health, prebiotics such as inulin and GOS may offer therapeutic potential for alleviating depressive symptoms by modulating GM composition and promoting the synthesis of neurotransmitters, SCFAs, and anti-inflammatory responses [38]. In addition, dairy-based products containing proteins, GOS, vitamins, and minerals may have the potential to improve sleep quality in individuals with sleep disturbances by modulating the GM, particularly through the promotion of beneficial bacteria such as *Bifidobacterium* [39].

Synbiotics consist of a combination of probiotics and prebiotics in which the prebiotic component enhances the viability of the probiotic, serves as a fermentable fiber source, and exerts its own prebiotic effects [40]. Although preclinical research has explored the potential of synbiotics in managing psychiatric symptoms, evidence on their application in human populations with mental health conditions remain scarce. Postbiotics, in turn, refer to the metabolic products generated through bacterial fermentation and encompass bioactive compounds (e.g., SCFAs) and molecules produced through host-microorganism interactions, including intestinal peptides [41]. Nevertheless, evidence regarding the administration of these bioactive molecules in humans and their impact on mental health is also currently limited. Table 1 (Ref. [37, 39, 42, 43, 44, 45, 46, 47, 48, 49, 50, 51, 52, 53, 54]) provides a summary of recent preclinical and clinical studies on psychobiotic administration for improving sleep.

**Table 1.** **Psychobiotic interventions for improving sleep**.

Supplementation	Treatment	Participants/Design	Outcomes	Reference
Preclinical studies				
PDX/GOS.	Prebiotic diet administration for 4 weeks.	Adult male Sprague Dawley rats (*N* = 96), 23-day-old, with sleep disruption (12 cohorts of 8 rats each). Longitudinal study.	The administration of prebiotics extended both NREM and REM sleep across five days of sleep disruption and enhanced total sleep duration during the 24-hour recovery period.	[42]
*Levilactobacillus brevis* strain ProGA28.	Daily probiotic supplementation for 2 weeks.	Wistar-Kyoto male rats (*N* = 8), 10–14 weeks old, exposed to cage exchange procedure. Longitudinal study.	Probiotic administration was found to alleviate stress-induced sleep disturbances, which appeared to be linked to enhanced parasympathetic activity and reduced anxiety-like behaviors.	[43]
*Limosilactobacillus fermentum *strain PS150.	Oral probiotic administration for 4 weeks.	Adult male C57BL/6J mice (*N* = 11), 6-week-old, exposed to cage change procedure and induced into sleep using pentobarbital. Longitudinal study.	Probiotic administration effectively reduced sleep latency and the time required to restore normal REM sleep levels. In addition, it significantly improved sleep disturbances induced by the FNE.	[44]
Test diet supplemented with prebiotics (GOS + PDX + lactoferrin + milk globules).	Oral prebiotic administration for 7 weeks.	Adult male F344 rats (*N* = 52), 24-day-old, exposed to an acute stressor (100, 1.5 mA tail shocks). Longitudinal study.	Dietary prebiotics prevented the stress-induced decrease in microbial alpha diversity. Several novel fecal metabolites were associated with sleep physiology, with pyrimidine nucleotide found to enhance NREM sleep.	[45]
Test diet supplemented with prebiotics (GOS + PDX + lactoferrin + milk globules).	Oral prebiotic administration for 13 weeks.	Adult male Sprague Dawley rats (*N* = 84), 23-day-old, exposed to CDR (12-hour light/dark reversal, weekly for 8 weeks). Longitudinal study.	Rats exposed to CDR while consuming a prebiotic diet, compared to a control diet, realigned NREM sleep and core body temperature diurnal rhythms to the altered light/dark cycle more quickly (ClockLab). The prebiotic diet led to rapid and sustained increases in the relative abundances of *Parabacteroides distasonis* and *Ruminiclostridium 5*.	[46]
Clinical studies				
Cow’s milk-based infant formula added prebiotic PDX and GOS.	Oral prebiotic administration for 70–112 days.	Healthy infants (*N* = 161), aged 14 to 35 days. Double-blind, RCT. Parallel-group, prospective study.	Infants receiving prebiotics showed faster consolidation of the daytime waking state, and supported the use of home-based actigraphy for assessing early sleep-wake patterns.	[47]
NVP-1704 probiotic mixture:* Limosilactobacillus reuteri* strain NK33 + *Bifidobacterium adolescentis* strain NK98.	Oral probiotic administration of NVP-1704 for 8 weeks.	Healthy adults (*N* = 122), 19–65 years old, exhibiting psychological stress and subclinical symptoms of depression and anxiety. Double-blind, RCT. Parallel-group, longitudinal study.	The NVP-1704 group exhibited a significant reduction in depressive symptoms at 4 and 8 weeks of treatment, and in anxiety symptoms at 4 weeks, compared to the placebo group. In addition, the NVP-1704 group showed an improvement in sleep quality.	[48]
*Bifidobacterium longum* strain AH1714.	Oral probiotic administration for 8 weeks.	Healthy male adults (*N* = 30), aged 18–30 years, enrolled at University College Cork. Double-blind, RCT. Crossover, longitudinal study.	Overall sleep quality and sleep duration improved significantly in the probiotic-treated group during exam stress, compared to the placebo-treated group.	[49]
Synbiotic: *Lacticaseibacillus rhamnosus* strain CNCM I-4036, *Bifidobacterium animalis* subsp. *lactis *strain CBP-001010, and* B. longum* strain ES1 + FOS.	Oral synbiotic or placebo administration for 30 consecutive days.	Male participants, including professional soccer players (*N* = 13) and sedentary students (*N* = 14). Triple-blinded, RCT. Crossover, longitudinal study.	The synbiotic intervention improved objective physical activity, sleep quality, and perceived general health, as well as reducing stress and anxiety levels, but only in the athlete group.	[50]
Resistant dextrin.	10 g per day prebiotic administration for 8 weeks.	Female adults with obesity and type 2 diabetes (*N* = 76), aged 30–65 years. Double-blind, RCT. Longitudinal study.	Prebiotic supplementation improved sleep quality and overall life satisfaction. It also led to a significant reduction in endotoxin levels, glycosylated hemoglobin, and pro-inflammatory/anti-inflammatory biomarkers, including IL-18, IL-6, TNF-α, and IL-10.	[51]
Dairy-based product containing protein, GOS, vitamins, and minerals.	Oral prebiotic administration for 3 weeks, followed by a 3-week washout period.	Healthy adults with sleep disturbances (*N* = 70), aged 30–50 years. Double-blind, RCT. Crossover, longitudinal study.	Compared to placebo (skimmed milk), PSQI was only significantly lower on day 14 of the second intervention period in the intention-to-treat analysis. Prebiotic supplementation reduced salivary cortisol levels and stimulated *Bifidobacterium*, which may play a role in improving sleep quality.	[39]
Regular yogurt: *Lactobacillus delbrueckii *subsp.* bulgaricus* and *Streptococcus thermophilus*. Probiotic yogurt: *B. animalis* subsp. *lactis* and *Lactobacillus acidophilus*.	The intervention group received 100 g of yogurt containing probiotics, while the control group received 100 g of regular yogurt daily for 6 weeks.	Postmenopausal women (*N* = 66), aged 45–55 years. Triple-blind, RCT. Longitudinal study.	There was no statistically significant difference between the two groups in terms of mean scores for depression and sleep quality.	[52]
Synbiotic ice-cream containing *L. acidophilus* strain LA-5, *Bifidobacterium animalis* strain BB-12 as probiotics and inulin as prebiotic.	Oral synbiotic administration for 30 days.	Military personnel (*N* = 65), aged 18–22 years. Double-blind, RCT. Parallel-group, longitudinal study.	Improved tenseness and sleepiness were observed in healthy young military personnel undergoing a 5-day field training.	[53]
*L. acidophilus* strain DDS-1 and *B. animalis* subsp. *lactis* strain UABIa-12.	Oral probiotic administration for 14 days.	Night shift workers (*N* = 87), aged 18–65 years. Double-blind, RCT. Parallel-group, longitudinal study.	Probiotics may help mitigate the effects of anticipatory stress on the immune system prior to night shifts.	[54]
*L. plantarum* strain JYLP-326.	Oral probiotic administration twice per day for 3 weeks.	College students with anxiety (*N* = 60). Double-blind, RCT. Parallel-group, longitudinal study.	Probiotic administration could alleviate symptoms of anxiety, depression, and insomnia in test-anxious students.	[37]

PDX, polydextrose; GOS, galactooligosaccharides; NREM, non-rapid eye movement; REM, rapid eye movement; FNE, first night effect; CDR, chronic disruption of rhythms; PSQI, Pittsburgh Sleep Quality Index; RCT, randomized controlled trial.

In recent years, FMT has gained attention as a therapeutic approach targeting the GM. This procedure involves transferring fecal material from a healthy donor into the gastrointestinal tract of a recipient, with the goal of achieving a durable and significant restoration of the recipient’s microbial community [55]. FMT has shown promising efficacy in improving sleep quality and alleviating symptoms of chronic insomnia, as well as positively affecting anxiety and depression, likely through improvements in GM composition, particularly an increase in beneficial bacteria such as *Bifidobacterium* and *Lactobacillus* [55]. In addition, washed microbiota transplantation (i.e., a process similar to FMT, but with enhanced safety and quality control) has also shown promising results in improving sleep quality and overall life quality in patients affected by sleep disorders, with significant improvements in sleep latency and total sleep time [56]. Table 2 (Ref. [55, 56, 57, 58, 59, 60, 61]) provides a summary of recent preclinical and clinical studies on FMT administration for improving sleep.

**Table 2.** **FMT interventions for improving sleep**.

Donors	Recipients	Procedure/Design	Outcomes	Reference
Non-insomnia individuals (*N* = 16).	Patients with chronic insomnia (*N* = 17).	The nasoduodenal route was used to administer 600 mL of donor fecal slurry. Observational, parallel pre-post design, longitudinal study.	FMT significantly improved the ISI, PSQI, SAS, and SDS scores, as well as the quality of life in chronic insomnia patients (76.47%) after 3 weeks of treatment. In these patients, the relative abundance of *Eggerthella* was notably higher at baseline, while the relative abundance of *Lactobacillu*s, *Bifidobacterium*, *Turicibacter*, *Anaerostipes*, and *Eisenbergiella* significantly increased following FMT treatment. The latter changes were positively correlated with the effectiveness of the treatment.	[55]
Healthy individuals (*N* = 23).	Patients with sleep disorders (*N* = 40).	WMT was administered via endoscope. Observational, follow-up design with multiple time points, longitudinal study.	WMT significantly improved sleep quality, including sleep latency and sleep duration, in patients with sleep disorders both in the short and medium term. The improvements in sleep quality and latency were also more pronounced with an increased number of treatment courses, with the effects of multiple treatment courses surpassing those of single and double treatment courses.	[56]
C57Bl/6J mice exposed to IH or RA for 6 weeks, with fecal matter collected and frozen.	C57Bl/6J naïve mice.	Oral gavage of fecal slurry was administered into naïve mice. Longitudinal study.	FMT-IH and FMT-RA mice displayed distinct taxonomic profiles, reflecting the previous effects of IH on the GM. In addition, FMT-IH mice showed increased sleep duration and a higher frequency of longer sleep bouts during the dark cycle, indicating enhanced sleepiness (*p * < 0.001 compared to FMT-RA mice).	[57]
Adult C57BL/6 male mice subjected to AL, IIF, or AIF between days 41–43.	Normal adult C57BL/6 male mice subjected to focal ischemia.	Oral gavage of fecal slurry was administered into normal mice. Longitudinal study.	FMT from the IIF or AIF cohorts had no significant impact on post-ischemic recovery of motor and cognitive function, nor on anxiety- or depression-like behaviors, when compared to FMT from the AL cohort. In addition, FMT from the IIF or AIF cohorts did not affect post-ischemic infarct volume, atrophy volume, or white matter damage.	[58]
Healthy individuals (*N* = 30).	Post-acute COVID-19 syndrome patients with insomnia (*N* = 60), assigned to FMT (*n* = 30) or control group (*n* = 30).	Administrated via EGD and FS. Non-randomized, open-label, prospective interventional study.	At week 12, a higher percentage of patients in the FMT group achieved insomnia remission compared to the control group (37.9% vs. 10.0%). The FMT group exhibited a significant reduction in ISI, PSQI, GAD-7, and ESS scores, along with a decrease in blood cortisol levels from baseline to week 12, whereas no significant changes were observed in the control group. In addition, FMT led to an enrichment of bacteria such as *Gemmiger formicilis* and a depletion of microbial pathways involved in the production of menaquinol derivatives.	[59]
Human patients diagnosed with insomnia.	GF BALB/c mice.	Oral gavage of human fecal slurry into GF mice. Longitudinal study.	Insomnia patients exhibited lower serum butyrate levels and a deficiency of butyrate-producing species in their GM. When the GM from insomnia patients was transplanted into GF mice, it induced insomnia-like behaviors, along with a reduction in serum butyrate levels. However, the oral administration of butyrate successfully alleviated sleep disturbances in the recipient mice.	[60]
Samples from a non-profit universal fecal microbiota bank.	Patients with poor sleep quality (*N* = 52).	WMT via TET. Observational, prospective study.	The scores for the five components of the PSQI (i.e., subjective sleep quality, sleep latency, sleep duration, habitual sleep efficiency, and sleep disturbances) decreased in patients with poor sleep quality. Baseline sleep duration scores were found to be an independent predictor of sleep improvement one month after WMT in these patients. Those who experienced sleep improvement also showed greater reductions in depression and IBS severity compared to patients who did not experience sleep improvement.	[61]

ISI, Insomnia Severity Index; PSQI, Pittsburgh Sleep Quality Index; SAS, Self-Rating Anxiety Scale; SDS, Self-Rating Depression Scale; AIF, fasting during nighttime; AL, fed ad libitum; IIF, fasting during daytime; GAD-7, Generalized Anxiety Disorder-7 Scale; ESS, Epworth Sleepiness Scale; IBS, Irritable Bowel Syndrome; IH, intermittent hypoxia; RA, room air; GF, germ-free; WMT, washed microbiota transplantation; EGD, esophagogastroduodenoscopy; FS, flexible sigmoidoscopy; TET, transendoscopic enteral tubing; FMT, fecal microbiota transplantation.

The growing use of FMT in both practice and clinical trials has created a demand for a greater supply of long-term available fecal donors and the development of donor screening programs that are both suitable and effective. As shown in Table 2, the donor screening criteria and fecal processing methods used in the included studies vary considerably, which introduces challenges for reproducibility and consistency. In addition, a variety of factors could affect the outcomes of these studies, such as the influence of the particular donor’s GM composition or the mode of delivery, which remain unexplored and leave gaps in our understanding of how FMT may affect sleep quality. Moreover, the current evidence is limited by a lack of large-sample, placebo-controlled studies. As a result, it is difficult to exclude the potential placebo effect on the observed improvements in sleep quality, complicating the validation of FMT as a therapeutic intervention for sleep disorders. Consequently, the widespread clinical application of FMT for sleep disorders may be premature without further exploration into its mechanisms and robust clinical validation.

## Discussion

Circadian rhythms regulate various biological systems, aligning them with environmental cycles, with sleep serving as a core component for health. In this context, the GM exhibits circadian fluctuations primarily driven by feeding and fasting cycles, influencing both host circadian rhythms and metabolic processes [17]. Evidence suggests that sleep deprivation, fragmentation, and circadian disruption (i.e., any disturbance or dysregulation that negatively impacts circadian function) may impact GM composition. However, while data from some studies do not support a causal role of the GM in sleep disturbances [62], other MR studies have established a causal relationship [24]. Thus, sleep appears to have a predominant influence on the GM, with sleep loss potentially inducing stress that disrupts the GM, thereby contributing to dysbiosis and to a range of metabolic and systemic disorders unrelated to gut health.

GM diversity, encompassing bacterial richness and evenness, has been associated with better sleep outcomes, such as higher sleep efficiency, longer total sleep time, and reduced WASO [18]. However, research also reports minimal effects of short-term sleep disruptions on GM diversity, suggesting that transient changes in sleep may not alter microbial composition, while long-term sleep patterns and regularity could influence GM diversity [19]. Certain bacterial strains related to inflammation, such as *Clostridiaceae*, *Oscillospiraceae*, *Proteobacteria*, and *Klebsiella*, have been found to be significantly modified in relation to sleep parameters, including the Bacillota/Bacteroidota ratio [25]. In addition, IL-6 has been associated with poor sleep quality, as higher levels of IL-6 are linked to shorter durations of slow-wave sleep and greater daytime sleepiness [63]. Nevertheless, the precise mechanisms linking IL-6, GM diversity, and sleep remain to be fully understood.

Desynchronization of circadian rhythms has been associated with GM dysbiosis, which in turn has been linked to increased risks of metabolic disorders such as obesity, insulin resistance, and type 2 diabetes [64]. Human research on sleep fragmentation has shown declines in beneficial gut bacteria, such as *Lactobacillus* and *Bifidobacterium*, as well as in SCFAs, such as acetate, propionate, and butyrate, which are linked to disrupted sleep quality and increased dysbiosis [65]. In turn, microbial activity influences sleep regulation by producing metabolites such as SCFAs, 5-HT, and GABA, which modulate sleep quality and health through mechanisms such as serotonergic signaling, vagal stimulation, and immune regulation [66]. Moreover, GM metabolites derived from Trp and dietary fiber play a significant role in regulating sleep and may contribute to sleep disturbances [67]. In this respect, probiotic interventions have been shown to improve sleep quality, likely via anti-inflammatory effects [65]. These findings highlight a potential bidirectional relationship between circadian rhythms, the GM, and sleep quality, suggesting that probiotic interventions may offer a promising approach to improve sleep and mitigate dysbiosis. However, further research is needed to fully understand the underlying mechanisms and therapeutic potential.

Current treatment of sleep disorders relies predominantly on pharmacotherapy. However, most approved agents are associated with a high incidence (>2%) of adverse effects across multiple systems, including neurological (e.g., somnolence, dizziness, memory impairment), gastrointestinal (e.g., nausea, diarrhea, dyspepsia), psychiatric (e.g., anxiety, depressive symptoms), respiratory (e.g., nasopharyngitis), and musculoskeletal aversive symptoms [68]. To avoid these adverse effects, increasing interest has been directed toward GM-targeted interventions. In this context, the emerging field of nutritional psychiatry may play a pivotal role, as it focuses on how specific dietary patterns and nutrient-derived compounds influence mental health outcomes, primarily integrating diet-based strategies with mental health interventions [69]. Indeed, evidence suggests that customized dietary modifications and GM-targeted approaches may complement established therapies such as pharmacotherapy or psychotherapy [69]. However, it is important to note that optimal intervention design should be guided by biomarkers (e.g., GM composition, inflammatory cytokines, nutrient status, genomic profiles) to clarify mechanistic links between diet, gut-brain interactions, and mental health outcomes [69]. Within the field of nutritional psychiatry, nutraceuticals are increasingly recognized as promising tools [69]. For instance, research in particular clinical settings has shown that cannabidiol (CBD) could be effective in improving sleep quality and reducing symptoms of anxiety and stress [70]. In this respect, research indicates that CBD supplementation can lead to notable improvements in sleep duration and quality, with evidence suggesting that it may be more effective than traditional treatments such as amitriptyline [70]. Although specific studies on CBD and the GM are limited, research on cannabis suggests that the endocannabinoid system can influence GM composition, including alterations in bacterial diversity and the abundance of SCFA-producing bacteria such as *Bifidobacterium*, *Coprococcus*, and *Faecalibacterium* [70].

Building on the aforementioned, it should be noted that the impact of diet on the microbial-circadian communication network could be substantial, as dietary patterns directly influence both circadian rhythms and the GM. Although most chrono-nutrition research has focused on the temporal aspects of diet, such as the timing, frequency, and regularity of food intake, the association between diet quality and circadian rhythms has also been explored [64]. In this respect, the relationship between diet and sleep is unclear, with limited evidence suggesting that chronotype may influence diet quality. Specifically, evening chronotypes tend to consume more sucrose, sweet foods, alcohol, and caffeine, while morning chronotypes show higher adherence to healthier diets, such as the Mediterranean diet, and have better health outcomes, such as lower waist circumference and visceral fat [64]. In addition to diet, several mental health conditions, particularly stress, anxiety, and depression, have been linked to sleep disorders, especially those stemming from circadian rhythm imbalances [4, 44]. Indeed, a higher risk of depression has been reported in individuals with evening chronotype compared to morning chronotype individuals [64]. This phenomenon is likely attributed to disrupted rhythmic activity in neurotransmitter systems involved in mood regulation, including dopamine and serotonin secretion. Consequently, diet and mental health conditions could act as confounding factors in the relationship between sleep and the GM. For instance, stress or depression may simultaneously cause sleep disturbances and alter the GM [4, 23], thereby acting as a potential confounder in the sleep-microbiota association. Similarly, high sugar intake, poor dietary patterns, or diet-induced conditions such as obesity may exacerbate both sleep disturbances and GM dysbiosis. Thus, without controlling for these factors, it becomes difficult to establish causality in the observed relationships between sleep and the GM. To better address these challenges, future clinical studies should incorporate more rigorous designs to control for confounding factors through randomized controlled trials and stratification into subgroups based on key variables such as dietary patterns and mental health comorbidities.

Currently, questions regarding the role of the GM in sleep disorders are not easily answered. For instance, whether the GM is a direct cause of sleep disorders or whether GM interventions are more advantageous than traditional treatments for sleep disorders might depend on several factors, including the specific condition being addressed, as well as its severity and duration, the individual characteristics of the patient, the specific underlying mechanisms involved, and the potential impact of comorbidities. Regarding the first question, emerging evidence suggests that the GM may play a role in sleep disorders, possibly contributing to a bidirectional relationship. In this respect, sleep disturbances could potentially lead to changes in the GM, while dysbiosis of the GM may exacerbate or even trigger sleep disturbances. Regarding the second question, the available evidence indicates that, in certain contexts, GM-targeted therapies could offer promising benefits, possibly with fewer side effects than conventional treatments. However, these interventions are still considered complementary and traditional approaches continue to be recommended as the first-line treatment for sleep disorders.

This review may present several limitations that should be appropriately acknowledged. First, the duration of most available clinical protocols is relatively short, allowing only speculative conclusions regarding long-term interactions between sleep and the GM. Extended follow-up studies are needed to identify stable GM-derived biomarkers associated with specific sleep parameters. Second, the mechanistic pathways through which gut microorganisms influence sleep remain insufficiently defined. Potential routes include direct neural signaling via the vagus nerve, as well as indirect modulation through endocrine, metabolic, and immune pathways. The development of molecular tools capable of real-time tracking of gut-brain communication will be essential to clarify these mechanisms. Third, observed differences in microbial composition may not be uniquely attributable to sleep-related variables, as they are confounded by genetics, sex, diet, physical activity, and drug use (particularly antibiotics). Fourth, the heterogeneity of sleep assessment methods, including self-report measures, limits comparability across studies and may introduce bias. Fifth, much of the current evidence is derived from cross-sectional studies, which hinder inferences about directionality or causality. Well-designed intervention studies are therefore required to establish temporal relationships between sleep disturbances and GM alterations. Sixth, most studies focus exclusively on α-diversity metrics, with limited reporting of β-diversity, thereby restricting evaluation of specific microbial taxa or species that may confer resilience or susceptibility to sleep disorders. Finally, it is essential to note that the associations presented in this review are derived from specific studies and may not be consistent across different populations or methodologies. Consequently, conclusions should be interpreted with caution and future research should aim to confirm the robustness and generalizability of the findings.

## Conclusions

Through its effects on bacterial metabolites, immune responses, and neuronal signaling, the GM might be potentially involved in the regulation of sleep-wake cycles. Disturbances in sleep have been associated with shifts in GM composition, but this relationship remains incompletely understood and it suggests a bidirectional nature. Advances in microbial sequencing and cultivation techniques may help clarify the role of specific gut bacteria in different sleep phases, although translating these findings into clinical treatments is still challenging. Evidence suggests that GM-targeted interventions, such as the administration of psychobiotics and FMT, may have potential for improving sleep outcomes, but further research is needed to determine their actual effectiveness. Understanding the full range of factors influencing the GM and their interactions with individual and external variables will be essential for elucidating the mechanisms behind gut-sleep interactions. Future research should focus on several key areas: (i) establishing associations through multimodal assessments, including neuroimaging, sleep evaluations, microbiome profiling from fecal samples, and metabolomics analyses of blood, saliva, and urine; (ii) identifying biomarkers through multi-omics integration, supported by machine learning, to classify microbial signatures and functional pathways related to sleep disturbances, as well as to inform personalized diagnostics and therapies; (iii) establishing causality through FMT studies in animal models and human trials, using longitudinal designs and multiple sampling, to clarify the effects of sleep disorders on microbial composition and function; and (iv) developing GM-targeted interventions, such as the genetic engineering of gut microorganisms to produce specific metabolites or bioactive compounds for the potential management of sleep disorders.

In essence, the role of the GM in sleep disorders is not yet fully understood. While emerging evidence suggests a bidirectional relationship in which sleep disturbances may alter the GM and vice versa, the exact mechanisms remain unclear, and further clinical exploration is needed. Therefore, ongoing investigations should aim at clarifying causality, identifying key biomarkers, and developing GM-targeted interventions to establish effective therapeutic strategies.

## Data Availability

Not applicable.

## Abbreviations

ACh, Acetylcholine; CBD, Cannabidiol; CBT-I, Cognitive behavioral therapy for insomnia; DA, Dopamine; ENS, Enteric nervous system; FMT, Fecal microbiota transplantation; FOS, Fructooligosaccharides; GABA, γ-aminobutyric acid; GBA, Gut-brain axis; GM, Gut microbiome; GOS, Galactooligosaccharides; HPA, Hypothalamic-pituitary-adrenal; ICSD-3-TR, International Classification of Sleep Disorders; LPS, Lipopolysaccharide; MR, Mendelian randomization; NE, Norepinephrine; NREM, Non-rapid eye movement; RCT, Randomized controlled trial; REM, Rapid eye movement; OSA, Obstructive sleep apnea; PSQI, Pittsburgh Sleep Quality Index; 5-HT, Serotonin; SCFAs, Short-chain fatty acids; Trp, Tryptophan; WASO, Wake after sleep onset.
